# Allergen-Specific IL-5 Responses in Early Childhood Predict Asthma at Age Eight

**DOI:** 10.1371/journal.pone.0097995

**Published:** 2014-05-29

**Authors:** Christina Weber-Chrysochoou, Daniele Crisafulli, Andrew Stewart Kemp, Warwick John Britton, Guy Barrington Marks, for the CAPS Investigators

**Affiliations:** 1 Respiratory and Environmental Epidemiology, Woolcock Institute of Medical Research, Sydney, New South Wales, Australia; 2 Sydney Medical School, University of Sydney, Sydney, New South Wales, Australia; 3 Department of Respiratory Medicine, Liverpool Health Service Hospital, South Western Sydney Clinical School, University of New South Wales, Sydney, New South Wales, Australia; 4 Department of Immunology, Centenary Institute of Cancer Medicine and Cell Biology, Sydney, New South Wales, Australia; 5 Department of Allergy and Immunology, Children’s Hospital Westmead, Sydney, New South Wales, Australia; National Jewish Health, United States of America

## Abstract

**Background:**

The pattern of development of allergen-specific T cell cytokine responses in early childhood and their relation to later disease is poorly understood. Here we describe longitudinal changes in allergen-stimulated T cell cytokine responses and their relation to asthma and allergic disease during the first 8 years of life.

**Methods:**

Subjects with a family history of asthma, who were enrolled antenatally in the Childhood Asthma Prevention Study (public trials registration number ACTRN12605000042640), had skin prick tests, clinical evaluation for asthma and eczema, and *in vitro* assessment of T cell cytokine responses to HDM extract performed at ages 18 months (n = 281), 3 years (n = 349), 5 years (n = 370) and 8 years (n = 275). We measured interleukin (IL-) 13 at 3, 5 and 8 years, and IL-5, IL-10, and interferon-γ (IFN-γ), at 18 months, 3, 5 and 8 years by ELISA. A cohort analysis was undertaken. Independent effects of cytokine responses at each age on the risk of asthma and allergic outcomes at age 8 years were estimated by multivariable logistic regression.

**Results:**

HDM-specific IL-5 responses increased with age. HDM-specific IL-13 and IL-10 responses peaked at age 5 years. HDM-specific IL-5 responses at 3 years, 5 years and 8 years were significantly associated with the presence of asthma and atopy at 8 years. IL-13 responses at 3 years, 5 years and 8 years were significantly associated with atopy at 8 years, but this association was not independent of the effect of IL-5. Other HDM-specific cytokine responses were not independently related to asthma or eczema at 8 years.

**Conclusion:**

HDM-specific IL-5 responses at age 3 years or later are the best measure of T cell function for predicting asthma at age 8 years.

## Introduction

There is an association between the pattern and level of cytokine production and the presence of atopic disease in early life [1]. The nature of this association is complex, as is the sequence of events that leads to the development of asthma. The T cell-derived cytokine interleukin (IL)-5 is primarily responsible for the cascade of eosinophil activation, while IL-4 and IL-13 contribute to immunoglobulin E (IgE) production [2].

While the natural history of asthma and its relation to allergic responses from age 8–10 years into adulthood has been well studied [3], early life events play a critical role in determining the pattern of immune maturation and the development of allergic disease and particularly asthma [4]. However, current understanding of these events is limited by the nature of the available data. Many of the existing studies are cross-sectional [5], limited to an early age [6] or limited by missing information between infancy and school age [7]. Birth cohort studies, in which cytokine responses are monitored during the first several years of life, are necessary to gain an understanding of the immunological pathways to clinical outcomes in later life.

Knowledge of the development of allergen-specific T cell cytokine responses during early life and their relation to subsequent asthma will enable better understanding of the immunological events leading to the development of allergic disease and, hence, provide new insights for prevention and therapy of asthma and allergic disease. Therefore, the objectives of this study were (a) to describe age-related changes in HDM-specific cytokine production by PBMCs between 18 months and 8 years and (b) to establish the age at which the onset of HDM-specific cytokine production predicts atopy or asthma in 8 year old children.

## Materials and Methods

### Study Design

The study cohort comprised children who were prenatally enrolled in the Childhood Asthma Prevention Study (CAPS), a high-risk cohort who participated in a randomized-controlled trial designed to test the effectiveness of HDM avoidance and alteration of the dietary fatty acids for the prevention of asthma and allergic disease. The study has been described in detail elsewhere [8], (public trials registration number is ACTRN12605000042640, quoted www.anzctr.org.au/Trial/Registration/TrialReview.aspx?id=48). Here we present a cohort analysis of the data.

### Participants and Sample Size

Pregnant women whose unborn children were at high risk of developing asthma were recruited from antenatal clinics of 6 hospitals in Sydney. At completion of recruitment in January 2000, 616 pregnant women had been enrolled and parents had given written, informed consent. The study was approved by the ethic committees of the University of Sydney, the Children’s Hospital at Westmead, and the Western and South Western Sydney Area Health Services.

### Clinical Assessment

Children were assessed at ages 18 months, 3, 5 and 8 years. The evaluation included a questionnaire-based interview of parents about symptoms and diagnoses relevant to asthma and other allergic disease. Subjects were defined as having ever been diagnosed with asthma, if they reported a doctor’s diagnosis of asthma. Parents were asked if the child had ever been diagnosed with eczema or atopic dermatitis. An examination for eczema was also performed. Skin prick tests were conducted to ingested allergens (salmon, tuna, peanuts, cow’s milk and egg) and to inhaled allergens (house dust mite, grasses, cockroach, cat and *Alternaria*). At age 5 and 8 years spirometric function was measured before and after the administration of salbutamol 200 µg. At age 8 years we assessed non-specific airway hyperresponsiveness (AHR) by methacholine challenge. AHR was defined as PD_20_FEV_1_ (methacholine) <6.1 µmol. We also classified those who did not have a response to methacholine challenge, but had a ≥12% FEV_1_ bronchodilator response as having AHR.

Children with a parental report of doctor diagnosed asthma and any wheeze in the last 12 months were classified as having current asthma at that age. At age 18 months and 3 years the inclusion period for wheeze was extended to 18 months. At ages 5 and 8 years those who a >12% increase in FEV_1_ after bronchodilator together with recent wheeze were also classified as having asthma, even if they did not report a doctor’s diagnosis. Finally, at age 8 years, those with recent wheeze and AHR were also classified as having asthma, irrespective of a reported doctor’s diagnosis.

Eczema was defined as the presence of flexural eczema on inspection by the assessment nurse or by parental report of a history of itchy rash coming and going over 3 months or more, together with a history of seeking medical care for eczema and/or using steroid or emollient creams in the last 12 months.

Atopy was defined using skin prick tests (SPTs) to ingested and inhalant allergens as previously described [9]. Wheal sizes that were ≥2 mm at the age of 18 months, 3 years and 5 years, and ≥3 mm at 8 years to at least one of the tested allergens and > than the positive control were classified as positive.

### PBMC Stimulation and Cytokines

Blood was collected into lithium heparin-containing tubes at the time of the clinical assessments at ages 18 months, 3, 5 and 8 years and stored at room temperature until it was processed. 6 hours within blood collection peripheral blood mononuclear cells (PBMC) were separated by centrifugation on a Ficoll-Paque density gradient (Amersham Biosciences) and washed twice in phosphate buffered saline (PBS). The cells were resuspended at 10^6^ viable cells/ml in serum-free culture medium (AIM-V medium, GIBCO Invitrogen Corp, California, USA). One ml of PBMC was distributed in 24 well plates with (1) phytohaemaglutinin (PHA, Sigma-Aldrich Co, St. Louis. MO, USA) at 10 µg/ml, (2) aqueous sonicated extract of HDM (CSL Bioscience, Melbourne, Australia) at 50 µg/ml, or (3) medium alone, and incubated at 37°C with 5% CO2. The cell-free supernatants were collected after 48 hours and stored at −70°C. The released cytokines were measured by ELISA, as previously described [9], [10], the cytokine IL-5, IL-10 and IFN-gamma kits: BD OptEIA Human Sets, BD Biosiences Pharmingen, catalogue numbers 555202, 555157 and 555142 respectively; IL-13 kit: IL-13 Module Set: Bender MedSystems, Vienna, Austria, Catalogue number BMS231/3MST. The limits of detection of these assays were established as the mean of the lowest measurable calibration point for each assay. The cytokine response in PHA-stimulated culture was treated as the positive control and medium only culture as the negative control. Cytokine levels from the HDM cultures were adjusted for background by deducting either the cytokine level from the corresponding negative control culture or the detection limit, whichever was greater. Results for samples in which levels of all the cytokines in the PHA culture were less than 3 times the corresponding cytokine level in the negative control culture (or the detection limit for that cytokine, whichever was greater) were considered invalid for all cytokines for that sample. Cytokine concentrations of IL-5, IL-13, and IL-10 in the HDM stimulated PBMC were classed as non-responders (<10 pg/ml), low responders (10–50 pg/ml) and high responders (>50 pg/ml). IFN-γ responses were classed as non-responders (<50 pg/ml) or responders (>50 pg/ml) because the lower limit of detection for this assay was higher than for the other cytokines.

### Statistical Methods

The distribution of HDM-stimulated cytokine responses at each age was displayed graphically using a box-and-whiskers plot to show the median, interquartile range and the outlying observations. Cross-sectional and longitudinal associations were examined using contingency tables with cytokine responses classified into two or three levels as described above. Associations were tested by univariate analysis using the chi square trend test. For each cytokine the odds ratio for its association with each outcome was estimated with simultaneous adjustment for the other cytokines measured at the same age as well as for sex and for the two randomized interventions. These odds ratio were estimated using logistic regression. Logistic regression was also used to test for the interaction of both atopic status and the two randomised interventions with cytokine responses in their effect on asthma and eczema outcomes.

The findings are reported in accordance with the STROBE guidelines [11].

## Results

**Figure 1:**
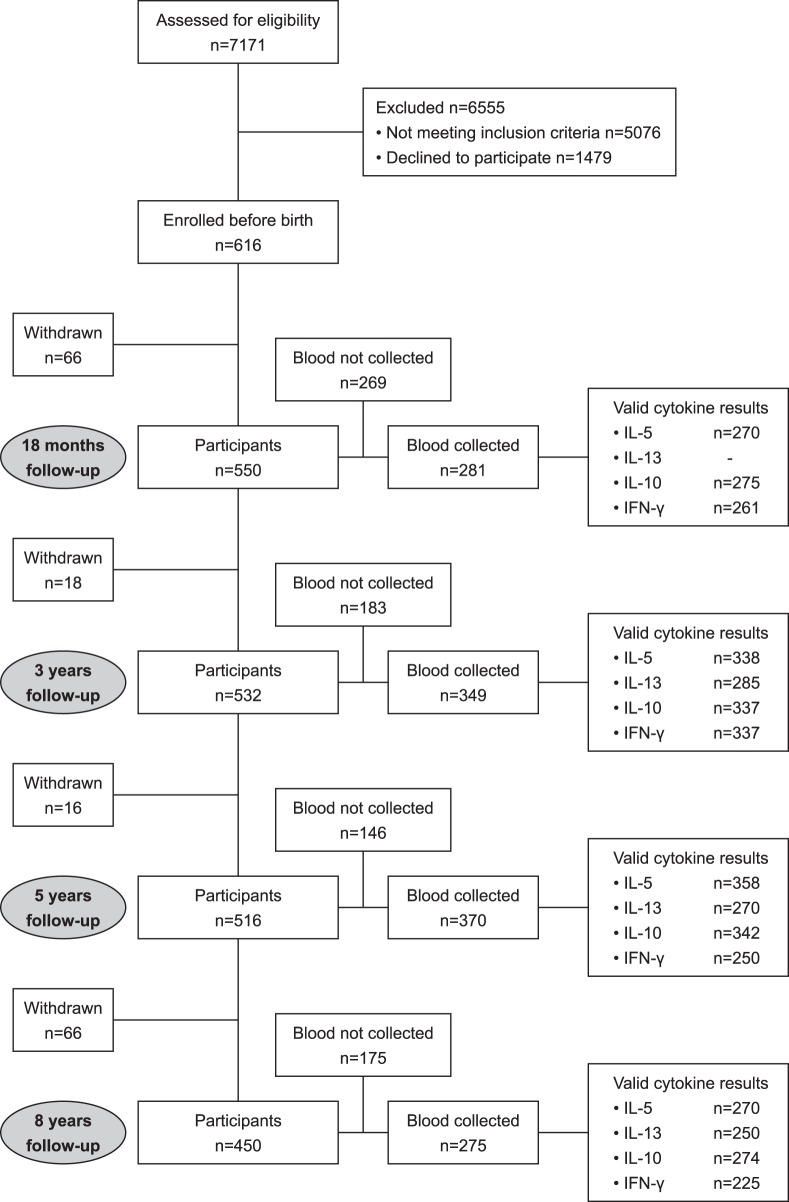
Flowchart for Childhood Asthma Prevention Study (followed to age 8 years). The size of the cohort at each assessment (18 months, 3 years, 5 years, 8 years): number of participants, number of blood samples, number of valid cytokine responses.

At age 8 years we examined 450 of the 616 children who had been enrolled antenatally, and collected blood from 316 of these. The derivation of this final cohort from the initially enrolled cohort is described in Figure 1. There were equal numbers of boys and girls in the study population (Table 1). The prevalence of asthma, atopy and eczema at each age is shown in Table 1.

**Table 1:** Characteristics of the study population at different ages.

	n (%) at 8 y	n (%) at 5 y	n (%) at 3 y	n (%) at 1.5 y
Male Sex	150 (50%)	184 (50%)	187 (54%)	127 (45%)
Active HDM intervention	139 (47%)	183 (49%)	171 (49%)	132 (47%)
Active Diet intervention	161 (54%)	191 (52%)	175 (50%)	137 (49%)
Atopy	131 (46%)	158 (43%)	103 (30%)	62 (22%)
Asthma	68 (23%)	78 (21%)	71 (20%)	32 (11%)
Eczema	36 (12%)	80 (22%)	81 (23%)	78 (28%)

### Pattern of HDM-specific Cytokine Responses Over the First 8 Years of Life

The number of valid assays for each cytokine at each age is shown in Figure 1. Table 2 shows the cytokine response profile of subjects at age 8 years. Data for the responses at earlier ages have been presented previously [9]. The prevalence of strongly positive HDM-specific IL-5 responses increased progressively with age. In contrast, the prevalence of strongly positive IL-13 responses reached a plateau at age 5 years, and the prevalence of strongly positive IL-10 responses also peaked at age 5 years but then declined by age 8 years. HDM-specific IFN-γ responses were uncommon at all ages.

**Table 2:** The frequency (n) of HDM-specific cytokine T-cell responses at age 8 years.

Category	IL-5	IL-13	IL-10	IFN-γ
<10 pg/mL	200	200	156	235
10–50 pg/mL	34	40	100	
>50 pg/mL	50	24	37	3

n: number of subjects.

### Cross-sectional Relationship between Cytokine Responses and Clinical Outcomes at Age 8 Years

Allergen-specific IL-5 responses at 8 years were strongly associated with the presence of asthma, eczema, atopy to any allergen and HDM-specific atopic responses, at this age (Table S1 in File S1 and Table S2 in File S1). IL-13 and IL-10 responses were not independently associated with asthma, eczema or atopy.

### Association of HDM-specific Cytokine Responses at all Ages and Clinical Outcomes at 8 Years

**Figure 2:**
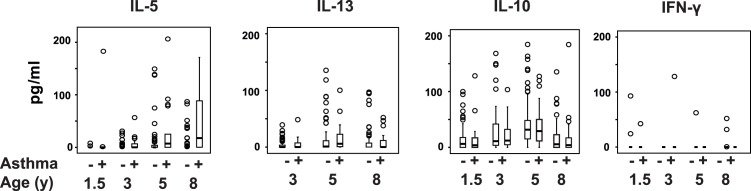
HDM-specific cytokine responses in asthmatic and non-asthmatic children. Box and whisker plots showing the distribution of HDM-specific IL-5, IL-13, IL-10 and IFN-γ cytokine responses at 18 months, 3, 5 and 8 years in asthmatic and non-asthmatic children at 8 years.

**Figure 3:**
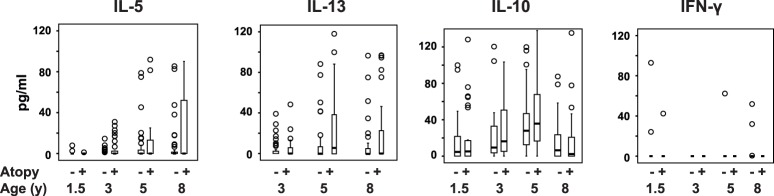
HDM-specific cytokine responses in atopic and non-atopic children. Box and whisker plots showing the distribution of HDM-specific IL-5, IL-13, IL-10 and IFN-γ cytokine responses at 18 months, 3, 5 and 8 years in atopic and non-atopic children at 8 years.

There was no association between HDM-specific T-cell responses at 18 months and the presence of asthma, atopy, or eczema at 8 years. From age 3 years HDM-specific IL-5 and IL-13 responses were associated with atopy and asthma at the age of 8 years (Figure 2 and 3). In the multivariate analysis, HDM-specific IL-5 responses at age 3, 5 and 8 years were each significantly associated with the presence of atopy and asthma at age 8 years (Table S3 in File S1). HDM-specific IL-13 responses at the age of 3 years, but not at 5 and 8 years, were significantly associated with asthma at 8 years. HDM-specific IL-13 responses at 3 years and at 5 years were significantly associated with atopy at 8 years. The associations between IL-5 responses and atopy to HDM were similar to the associations between IL-5 responses and atopy to any allergen.

After adjusting for the effect of other cytokine responses, sex and intervention groups, HDM-specific IL-5 responses at 5 years were still significantly associated with asthma at 8 years. However, IL-5 responses at 3 years were not associated with asthma at 8 years, and IL-13 responses at 3 and 5 years were not associated with asthma or atopy at 8 years, after adjustment for these same covariates. Other cytokine responses were not independently associated with these outcomes at age 8 years.

HDM-specific IL-5 and IFN-γ responses at age 3 years were independently and positively associated with the presence of eczema at age 8 years (Table 2).

The associations of HDM-stimulated cytokine responses at ages 18 months, 3 years, 5 years and 8 years with asthma at age 8 years was not significant modified by atopic status at the age when the cytokine responses were measured (P>0.1 in all cases). Also, these associations were not significantly modified by the randomised diet and HDM avoidance intervention groups (P>0.9 in all cases).

**Figure 4:**
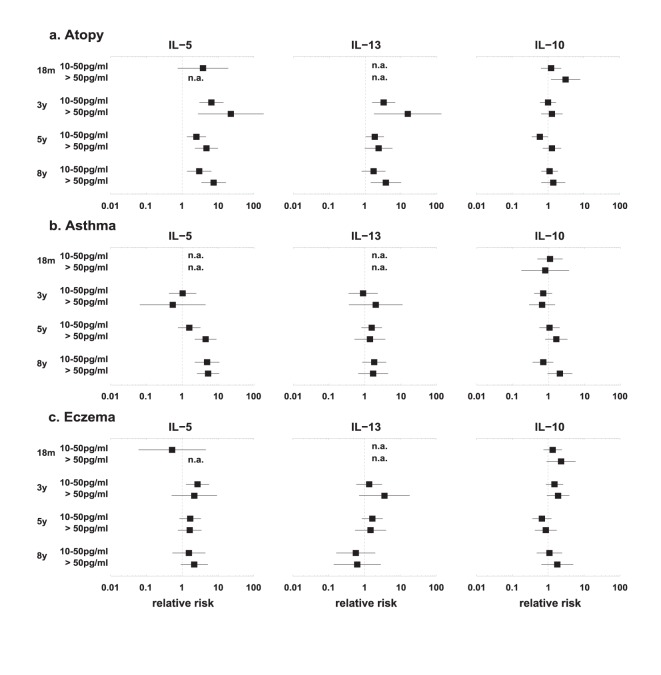
Relative risk of HDM-specific cytokine response and presence of atopy, asthma and eczema. The relative risk of HDM-specific IL-5, IL-13 and IL-10 responses at each time point and the presence of atopy (a), asthma (b) and eczema (c). Relative risks for low responders (10–50 pg/mL) and high responders (>50 pg/mL) compared with non-responders (<10 pg/mL) are shown with 95% CIs. Relative risks are not adjusted for other cytokines measured at the same age. n.a. = non applicable.

In the univariate analysis HDM-specific IL-5 and IL-13 responses were predictive for the presence of atopy at the age of 8 years from the age of three years. Figure 4 shows the relative risk of IL-5, resp. IL-13 at two response levels to get atopy (Figure 4a) and asthma (Figure 4b) at different ages. Also a high HDM-specific IL-5 response (>50 pg/ml) was predictive for the presence of asthma at the age 8 years (Figure 4b). At 18 months the number of high responders was too small to permit the determination of relative risk for this age group. There were low numbers of HDM-specific IFN-γ responders at all ages.

## Discussion

We have shown significant univariate associations between HDM-specific IL-5 and IL-13 responses at age 3 years, 5 years and 8 years and the presence of asthma and atopy at 8 years. When these responses were corrected for the responses of other cytokines in a multivariate model, only the HDM-specific IL-5 responses were significantly and independently associated with atopy and asthma at 8 years. Hence, HDM-specific IL-5 T cell responses are the best predictor for the presence of asthma and atopy at the age at 8 years. This association is evident from age three years, but not at age 18 months when there appears to be more plasticity in allergen-specific T cell responses [9].

The association of asthma with allergy to HDM, the dominant aeroallergen in Sydney [12], after the age of 8 years is well established [12]–[14]. Furthermore, the presence of atopy at age 7 to 8 years increases the likelihood of having more severe asthma in adulthood [15]. Our study, together with others, contributes to the understanding of the immunological events preceding the allergic manifestations and clinical associations described in these studies.

These findings have practical implications. IL-5 has an important role in the development and activation of eosinophils. The clinical significance of this finding is highlighted by the observation that therapy with an anti-IL-5 antibody has beneficial effects, including a reduction in exacerbation frequency, in a selected subgroup of adults with severe asthma [16], [17]. Furthermore, HDM avoidance may also be beneficial in improving asthma control in highly selected populations [18]–[20], in part through decreased T cell activation within the airways. Targeted molecular therapy, environmental interventions or other therapies could have a role in early secondary prevention if applied in specific high-risk populations. It is plausible that children with IL-5 responses to *in vitro* HDM stimulation at the age of 3 years might be the relevant target group for these interventions. This cohort was initially recruited into a randomized controlled trial of HDM avoidance [21]. Due to the small number of respondents with IL-5 responses to HDM stimulation at age 3 years, it was not possible to directly test the hypothesis that HDM avoidance was more effective at preventing asthma in this sub-group. Nevertheless, we do have indirect evidence of the plausibility of this approach. We have shown that the HDM avoidance intervention, implemented from birth to age 5 years, was effective in reducing the prevalence of poorly controlled asthma at age 8 years in those children who were sensitized to HDM at age three years [21].

We found very low IFN-γ levels after allergen stimulation, even at the age of 8 years, and there was no association between allergen-specific IFN-γ responses at 18 months or 3 years and subsequent allergic disease. The association between reduced IFN-γ responses and atopy or asthma has generally been characterised using polyclonal stimulation of T cells with mitogen [1], [22]. Van der Velden et al. did observe a difference between atopic and non-atopic children in IFN-γ and in IL-10 responses to specific (HDM) stimulation of cord blood mononuclear cells or PBMC collected at 12 months [23]. However, these investigators continued antigen stimulation for 7 days, which was substantially longer than the 48 hour period of stimulation employed in this study. In addition, we did not find any association between allergen-specific IL-10 responses and asthma or atopy at any age. Presumably the mechanism of action of this immunomodulatory cytokine is more complex than is detectable by testing for direct relationship with the presence of disease.

The absence of any association between cytokine responses at an early age and outcomes at age 8 years implies that other mechanisms are relevant to asthma symptoms before the age of 3 years. It is possible that cytokine responses to non-specific, polyclonal T cell stimulation are more relevant to the immunologic mechanism of eczema, as has been suggested by others [4].

The strengths of this study are the birth cohort design, followed for 8 years, the repeated measurement of cytokine responses and the well-characterised phenotypes in a large community-based study sample. One limitation of the CAPS cohort is that it is a selected population with a family history of asthma. Therefore we cannot exclude the possibility that the association between allergen-specific IL-5 responses and asthma and atopy is different in children without a family history of asthma. It would be interesting to investigate whether this sub-group of IL-5 responders might represent a risk group in whom HDM immunotherapy would be an effective preventive intervention, as shown in the PAT study for pollen allergy [24].

We conclude that allergen-specific cytokine responses have meaningful associations with disease manifestations from age 3 years. Prior to this age, symptoms presumably arise in other ways, unrelated to allergen-specific immunological mechanisms. Our findings have implications for understanding the biology of allergic disease in childhood and the identification of children for secondary prevention interventions and nature of those interventions.

## Supporting Information

File S1 **Tables S1–S3.** Table S1. Cross-sectional association of cytokine profiles with the presence of atopy, asthma or eczema to any allergen at 8 years. Table S2. Cross-sectional association of cytokine profiles with the presence of atopy to HDM at 8 years. Table S3. Longitudinal association of cytokine responses at different ages with the presence of atopy, asthma and eczema to any allergen at the age of 8 years. Odds ratio is shown as a point estimate and the 95% confidence limits. *p* ^1^-values are based on univariate analysis (Mantel-Haenszel Chi-Square), *p* ^2^-values are based on multivariate analysis adjusted for other cytokines.
(DOCX)
