# Supplementary material for: Allergen-Specific IL-5 Responses in Early Childhood Predict Asthma at Age Eight
Source: PLoS One. 2014 May 29;9(5):e97995. doi: 10.1371/journal.pone.0097995 (PMC4038510; doi:10.1371/journal.pone.0097995)
Supplement: File S1 — Tables S1–S3. Table S1. Cross-sectional association of cytokine profiles with the presence of atopy, asthma or eczema to any allergen at 8 years. Table S2. Cross-sectional association of cytokine profiles with the presence of atopy to HDM at 8 years. Table S3. Longitudinal association of cytokine responses at different ages with the presence of atopy, asthma and eczema to any allergen at the age of 8 years. Odds ratio is shown as a point estimate and the 95% confidence limits. p 1-values are based on univariate analysis (Mantel-Haenszel Chi-Square), p 2-values are based on multivariate analysis adjusted for other cytokines. (DOCX) [file pone.0097995.s001.docx]

**Table S1**

a. Atopy

| Cytokine | Response | Atopy | No atopy | Adjusted Odds Ratio (95% Confidence Interval) | *p*-value |
| --- | --- | --- | --- | --- | --- |
| IL-5 | Non responder | 63 | 118 |  | 0.008 |
|  | Low responder | 19 | 12 | 3.1 (0.9-10.4) |  |
|  | High responder | 36 | 9 | 7.6 (1.9-30.0) |  |
| IL-13 | Non responder | 76 | 108 |  | 0.96 |
|  | Low responder | 17 | 14 | 0.9 (0.3-2.7) |  |
|  | High responder | 16 | 6 | 0.8 (0.2-4.3) |  |
| IL-10 | Non responder | 62 | 77 |  | 0.20 |
|  | Low responder | 42 | 47 | 0.9 (0.4-2.1) |  |
|  | High responder | 17 | 15 | 0.3 (0.1-1.2) |  |
| IFN-γ | Non responder | 101 | 110 |  | 0.36 |
|  | Responder | 2 | 1 | 4.1 (0.2-81.0) |  |

b. Asthma

| Cytokine | Response | Asthma | No asthma | Adj. OR (95% CI) | *p*-value |
| --- | --- | --- | --- | --- | --- |
| IL-5 | Non responder | 26 | 162 |  | 0.0006 |
|  | Low responder | 15 | 19 | 4.9 (1.5-16.1) |  |
|  | High responder | 21 | 25 | 12.4 (3.1-49.7) |  |
| IL-13 | Non responder | 41 | 150 |  | 0.19 |
|  | Low responder | 12 | 24 | 0.5 (0.2-1.8) |  |
|  | High responder | 7 | 15 | 0.2 (0.04-1.1) |  |
| IL-10 | Non responder | 35 | 111 |  | 0.46 |
|  | Low responder | 17 | 76 | 0.6 (0.2-1.7) |  |
|  | High responder | 13 | 20 | 1.3 (0.4-4.9) |  |
| IFN-γ | Non responder | 51 | 169 |  | 0.77 |
|  | Responder | 1 | 2 | 0.6 (0.03-14.3) |  |

c. Eczema

| Cytokine | Response | Eczema | No eczema | Adj. OR (95% CI) | *p*-value |
| --- | --- | --- | --- | --- | --- |
| IL-5 | Non responder | 19 | 168 |  | 0.02 |
|  | Low responder | 5 | 29 | 2.4 (0.5-11.7) |  |
|  | High responder | 9 | 37 | 10.1 (2.0-52.3) |  |
| IL-13 | Non responder | 26 | 164 |  | 0.04 |
|  | Low responder | 3 | 33 | 0.2 (0.05-1.3) |  |
|  | High responder | 2 | 20 | 0.04 (0.003-0.5) |  |
| IL-10 | Non responder | 16 | 129 |  | 0.66 |
|  | Low responder | 11 | 82 | 1.5 (0.5-4.6) |  |
|  | High responder | 6 | 27 | 2.1 (0.4-12.4) |  |
| IFN-γ | Non responder | 27 | 193 |  | 0.85 |
|  | Responder | 1 | 2 | 1.5 (0.02-96.2) |  |

**Table S2**

Atopy to HDM

| Cytokine | Response | Atopy  to HDM | No atopy  to HDM | Adjusted Odds Ratio (95% Confidence Interval) | *p*-value |
| --- | --- | --- | --- | --- | --- |
| IL-5 | Non responder | 44 | 137 |  | 0.0005 |
|  | Low responder | 15 | 16 | 2.8 (0.8-10.0) |  |
|  | High responder | 33 | 12 | 27.3 (5.1- 144.1) |  |
| IL-13 | Non responder | 60 | 124 |  | 0.22 |
|  | Low responder | 12 | 19 | 0.3 (0.07-1.3) |  |
|  | High responder | 13 | 9 | 0.2 (0.04-1.55) |  |
| IL-10 | Non responder | 47 | 92 |  | 0.29 |
|  | Low responder | 33 | 56 | 0.6 (0.25-1.5) |  |
|  | High responder | 14 | 18 | 0.3 (0.08-1.4) |  |
| IFN-γ | Non responder | 75 | 136 |  | 0.74 |
|  | Responder | 1 | 2 | 0.58 (0.02-14.3) |  |

**Table S3**

1. Atopy

| Age | Cytokine | Response | Atopy | No atopy | *p*¹-value | Adj. OR | *p*²-value |
| --- | --- | --- | --- | --- | --- | --- | --- |
| 18 m | IL-5 | Non responder | 79 | 119 | 0.6 |  | 0.5 |
|  |  | Low responder | 3 | 3 |  | 1.7  (0.3-9.6) |  |
|  |  | High responder | 0 | 0 |  | Not estimable |  |
|  | IL-10 | Non responder | 55 | 82 | 0.3 |  | 0.04 |
|  |  | Low responder | 18 | 37 |  | 0.7  (0.4-1.4) |  |
|  |  | High responder | 10 | 4 |  | 3.8  (1.1-13.0) |  |
|  | IFN-γ | Non responder | 76 | 113 | 0.7 |  | n.a. |
|  |  | Responder | 2 | 4 |  | n.a. |  |
| 3 y | IL-5 | Non responder | 91 | 145 | < 0.0001 |  | 0.02 |
|  |  | Low responder | 22 | 3 |  | 7.1  (1.8-28.6) |  |
|  |  | High responder | 5 | 1 |  | 1.9  (0.2-23.8) |  |
|  | IL-13 | Non responder | 73 | 121 | 0.0002 |  | 0.7 |
|  |  | Low responder | 18 | 9 |  | 1.5  (0.5-4.4) |  |
|  |  | High responder | 4 | 0 |  | n.a. |  |
|  | IL-10 | Non responder | 53 | 77 | 0.1 |  | 0.6 |
|  |  | Low responder | 44 | 54 |  | 0.9  (0.5-1.8) |  |
|  |  | High responder | 21 | 17 |  | 1.5  (0.6-3.8) |  |
|  | IFN-γ | Non responder | 114 | 147 | 0.9 |  | 1.0 |
|  |  | Responder | 2 | 3 |  | 1.0  (0.2-6.9) |  |
| 5y | IL-5 | Non responder | 84 | 144 | < 0.0001 |  | 0.0035 |
|  |  | Low responder | 24 | 17 |  | 3.9  (1.3-11.4) |  |
|  |  | High responder | 25 | 8 |  | 17.3  (2.6-116.0) |  |
|  | IL-13 | Non responder | 66 | 94 | 0.005 |  | 0.6 |
|  |  | Low responder | 27 | 22 |  | 0.6  (0.3-1.6) |  |
|  |  | High responder | 13 | 5 |  | 0.6  (0.1-4.1) |  |
|  | IL-10 | Non responder | 37 | 44 | 0.5 |  | 0.8 |
|  |  | Low responder | 50 | 86 |  | 0.9  (0.4-1.9) |  |
|  |  | High responder | 36 | 34 |  | 0.7  (0.3-1.8) |  |
|  | IFN-γ | Non responder | 92 | 115 | 0.4 |  | 1.0 |
|  |  | Responder | 1 | 3 |  | n.a. |  |
| 8y | IL-5 | Non responder | 63 | 118 | < 0.0001 |  | 0.008 |
|  |  | Low responder | 19 | 12 |  | 3.1  (0.9-10.4) |  |
|  |  | High responder | 36 | 9 |  | 7.6  (1.9-30.0) |  |
|  | IL-13 | Non responder | 76 | 108 | 0.003 |  | 1.0 |
|  |  | Low responder | 17 | 14 |  | 0.9  (0.3-2.7) |  |
|  |  | High responder | 16 | 6 |  | 0.8  (0.2-4.3) |  |
|  | IL-10 | Non responder | 62 | 77 | 0.4 |  | 0.2 |
|  |  | Low responder | 42 | 47 |  | 0.9  (0.4-2.1) |  |
|  |  | High responder | 17 | 15 |  | 0.3  (0.1-1.2) |  |
|  | IFN-γ | Non responder | 101 | 110 | 0.5 |  | 0.4 |
|  |  | Responder | 2 | 1 |  | 4.1  (0.2-81.0) |  |

1. Asthma

| Age | Cytokine | Response | Asthma | No asthma | *p*¹-value | Adj. OR | *p*²-value |
| --- | --- | --- | --- | --- | --- | --- | --- |
| 18 m | IL-5 | Non responder | 47 | 173 | 0.3 |  | 1.0 |
|  |  | Low responder | 1 | 5 |  | 1.0  (0.1-9.1) |  |
|  |  | High responder | 1 | 0 |  | n.a. |  |
|  | IL-10 | Non responder | 36 | 117 | 0.4 |  | 0.5 |
|  |  | Low responder | 13 | 49 |  | 0.7  (0.3-1.5) |  |
|  |  | High responder | 2 | 13 |  | 0.5  (0.1-1.5) |  |
|  | IFN-γ | Non responder | 48 | 161 | 0.3 |  | n.a. |
|  |  | Responder | 0 | 8 |  | n.a. |  |
| 3 y | IL-5 | Non responder | 51 | 213 | 0.0002 |  | 0.08 |
|  |  | Low responder | 10 | 17 |  | 2.2  (0.7-7.2) |  |
|  |  | High responder | 5 | 2 |  | 13.0  (1.2-143.9) |  |
|  | IL-13 | Non responder | 40 | 177 | 0.014 |  | 0.5 |
|  |  | Low responder | 10 | 19 |  | 1.9  (0.6-5.6) |  |
|  |  | High responder | 2 | 2 |  | 0.7  (0.04-13.6) |  |
|  | IL-10 | Non responder | 34 | 112 | 0.9 |  | 0.2 |
|  |  | Low responder | 21 | 85 |  | 0.5  (0.3-1.2) |  |
|  |  | High responder | 11 | 34 |  | 0.5  (0.2-1.5) |  |
|  | IFN-γ | Non responder | 62 | 226 | 0.2 |  | 0.1 |
|  |  | Responder | 3 | 4 |  | 3.6  (0.7-17.9) |  |
| 5y | IL-5 | Non responder | 45 | 199 | < 0.0001 |  |  |
|  |  | Low responder | 15 | 31 |  | 3.4  (1.2-9.6) |  |
|  |  | High responder | 18 | 20 |  | 8.8  (1.7-44.4) |  |
|  | IL-13 | Non responder | 32 | 138 | 0.09 |  | 0.1 |
|  |  | Low responder | 20 | 34 |  | 1.8  (0.7-4.7) |  |
|  |  | High responder | 5 | 17 |  | 0.3  (0.1-2.1) |  |
|  | IL-10 | Non responder | 24 | 63 | 0.9 |  | 0.1 |
|  |  | Low responder | 28 | 121 |  | 0.4  (0.2-1.1) |  |
|  |  | High responder | 22 | 55 |  | 0.4  (0.2-1.2) |  |
|  | IFN-γ | Non responder | 49 | 175 | 0.01 |  |  |
|  |  | Responder | 3 | 1 |  | n.a. | 1.0 |
| 8y | IL-5 | Non responder | 26 | 162 | < 0.0001 |  | 0.0006 |
|  |  | Low responder | 15 | 19 |  | 4.9  (1.5-16.1) |  |
|  |  | High responder | 21 | 25 |  | 12.4  (3.1-49.7) |  |
|  | IL-13 | Non responder | 41 | 150 | 0.1 |  | 0.2 |
|  |  | Low responder | 12 | 24 |  | 0.5  (0.2-1.8) |  |
|  |  | High responder | 7 | 15 |  | 0.2  (0.04-1.1) |  |
|  | IL-10 | Non responder | 35 | 111 | 0.3 |  | 0.5 |
|  |  | Low responder | 17 | 76 |  | 0.6  (0.2-1.7) |  |
|  |  | High responder | 13 | 20 |  | 1.4  (0.4-4.9) |  |
|  | IFN-γ | Non responder | 51 | 169 | 0.7 |  | 0.8 |
|  |  | Responder | 1 | 2 |  | 0.6  (0.03-14.3) |  |

1. Eczema

| Age | Cytokine | Response | Eczema | No eczema | *p*¹-value | Adj. OR | *p*²-value |
| --- | --- | --- | --- | --- | --- | --- | --- |
| 18 m | IL-5 | Non responder | 24 | 183 | 0.004 |  | 0.4 |
|  |  | Low responder | 2 | 4 |  | 3.8  (0.6-23.8) |  |
|  |  | High responder | 1 | 0 |  | n.a. |  |
|  | IL-10 | Non responder | 20 | 123 | 0.8 |  | 0.5 |
|  |  | Low responder | 7 | 52 |  | 0.5  (0.2-1.6) |  |
|  |  | High responder | 2 | 13 |  | 0.8  (0.2-1.6) |  |
|  | IFN-γ | Non responder | 26 | 172 | 0.3 |  | n.a. |
|  |  | Responder | 0 | 6 |  | n.a. |  |
| 3 y | IL-5 | Non responder | 23 | 186 | 0.03 |  | 0.04 |
|  |  | Low responder | 5 | 24 |  | 4.3  (1.0-18.9) |  |
|  |  | High responder | 2 | 2 |  | 23.0  (1.6-321.4) |  |
|  | IL-13 | Non responder | 26 | 228 | < 0.0001 |  | 0.9 |
|  |  | Low responder | 9 | 18 |  | 0.8  (0.2-3.5) |  |
|  |  | High responder | 3 | 3 |  | 1.0  (0.05-18.5) |  |
|  | IL-10 | Non responder | 18 | 123 | 1.0 |  | 0.3 |
|  |  | Low responder | 15 | 88 |  | 0.6  (0.2-1.6) |  |
|  |  | High responder | 5 | 37 |  | 0.3  (0.07-1.6) |  |
|  | IFN-γ | Non responder | 33 | 245 | 0.02 |  | 0.03 |
|  |  | Responder | 3 | 4 |  | 6.8  (1.2-37.1) |  |
| 5y | IL-5 | Non responder | 28 | 212 | 0.01 |  | 0.3 |
|  |  | Low responder | 10 | 32 |  | 1.9  (0.6-7.0) |  |
|  |  | High responder | 9 | 28 |  | 3.4  (0.6-19.7) |  |
|  | IL-13 | Non responder | 19 | 150 | 0.07 |  | 0.5 |
|  |  | Low responder | 12 | 38 |  | 2.0  (0.6-6.1) |  |
|  |  | High responder | 4 | 18 |  | 1.2  (0.1-9.5) |  |
|  | IL-10 | Non responder | 11 | 74 | 0.7 |  | 0.06 |
|  |  | Low responder | 22 | 124 |  | 1.7  (0.6-4.4) |  |
|  |  | High responder | 11 | 62 |  | 0.3  (0.1-1.5) |  |
|  | IFN-γ | Non responder | 35 | 185 | 0.4 |  | 1.0 |
|  |  | Responder | 0 | 4 |  | n.a. |  |
| 8y | IL-5 | Non responder | 19 | 168 | 0.07 |  | 0.02 |
|  |  | Low responder | 5 | 29 |  | 2.4  (0.5-11.7) |  |
|  |  | High responder | 9 | 37 |  | 10.1  (2.0-52.3) |  |
|  | IL-13 | Non responder | 26 | 164 | 0.4 |  | 0.04 |
|  |  | Low responder | 3 | 33 |  | 0.2  (0.05-1.3) |  |
|  |  | High responder | 2 | 20 |  | 0.04  (0.003-0.5) |  |
|  | IL-10 | Non responder | 16 | 129 | 0.3 |  | 0.7 |
|  |  | Low responder | 11 | 82 |  | 1.5  (0.5-4.6) |  |
|  |  | High responder | 6 | 27 |  | 2.1  (0.4-12.4) |  |
|  | IFN-γ | Non responder | 27 | 193 | 0.3 |  | 0.9 |
|  |  | Responder | 1 | 2 |  | 1.5  (0.02-96.2) |  |
